# Consequences of Disregarding Metric Invariance on Diagnosis and Prognosis Using Psychological Tests

**DOI:** 10.3389/fpsyg.2018.00167

**Published:** 2018-02-15

**Authors:** David Blanco-Canitrot, Jesús M. Alvarado, Daniel Ondé

**Affiliations:** Department of Psychobiology - Behavioral Sciences Methods & Institute of Biofunctional Studies from Complutense University of Madrid, Madrid, Spain

**Keywords:** invariance, differential item functioning, predictive value of tests, reliability and validity

## Introduction

Guenole and Brown (2014) have shown how failure to meet invariance criteria affects to path coefficients in SEM. In applied research context, these authors suggest testing non-invariance to detect possible undesired effects in the subsequent model evaluation. According to this line of argument, this work intends to show the negative consequences of ignoring the property of invariance when a scale is used with selection or diagnostic purposes.

A scale is invariant when subjects from different groups with the same level on the latent variable have the same probability of obtaining equal test score. However, invariance is not an all-or-nothing judgment. In multi-group Confirmatory Factor Analysis (CFA), four levels of invariance are defined (Meredith, 1993): configural invariance (prerequisite of same factorial structure), metric invariance (MI) or weak invariance (equality of factor loadings), scalar or strong invariance (equality of factor loadings and intercepts), and strict invariance (equality of factor loadings, intercepts and residuals). When a multi-group CFA is conducted, the evaluation of these types of invariance consists on a stepwise procedure from the least restrictive solutions (configural vs. MI) to the most restrictives (MI vs. strong and strong vs. strict), using nested χ^2^ tests (Brown, 2015). Consequently, the evaluation of MI is a necessary requirement to compare group scores (Millsap, 2011).

In the parallel model of Classical Test Theory (CTT), MI is directly related to reliability^1^. In this model all items have the same standardized factor loading (λ), and the communality (λ^2^) is equal to the average correlation of the scale. Consequently, for a scale of *n* items, reliability of a given value of λ can be calculated from the standardized alpha coefficient: α = *n*λ^2^ / (1 + (*n* − 1)λ^2^).

Relationship between reliability and predictive validity was first established by Gulliksen (1950) and his attenuation formula. However, the effect of loss of reliability in one of the groups of the sample over the predictive validity is not sufficiently known. What happens when discriminability of some items (i.e., their factor loadings) is different between groups and the instrument is used to make predictions on a dichotomous pass/fail test criterion? How can this MI problem interfere with the correct classification of subjects? This paper aims to explore common practices in applied research that usually ignore MI evaluation (Borsboom, 2006). In this paper, we will try to show the need to reconsider the practical usefulness of psychological tests and scales in decision-making, due to the biased in the correct classification of the subjects.

## Methods

### Simulation procedure

Common values in applied research of reliability and sample size were simulated via Monte Carlo study with 500 sample replications (Harwell et al., 1996) of 100 statistical units for each group (*N* = 200), where factor loadings of a ten-item scale were simulated between 0.44 and 0.50, with an associated reliability of 0.71 and 0.77. In applied psychology, the median sample size of non-students is 200 (Shen et al., 2011). There are between 1 and 10 items per scale in more than 90% of the studies (Hinkin, 1995). To reach Nunnally (1978) recommendation regarding reliability in applied research contexts (minimum of 0.70), for a 10-item scale, a factor loading of 0.44 per item is needed. Following the parallel model, α = 10(0.44)^2^ / (1+9(0.44)^2^) = 0.706.

The database was generated based from the factorial model that is defined in Equation (1).

(1) $$X_{ij}=\sum_{k=1}^{k}\lambda_{jk}F_{k}+\sqrt{\left(1-\sum_{k=1}^{k}\lambda_{jk}^{2}\right)}\times e_{j}$$

Where *X*_*ij*_ is the simulated response of subject *i* on a given item *j*, λ_*jk*_ is the loading item *j* in a factor *k* (which was generated by an unifactorial model), *F*_*k*_ is the latent factor generated by a standardized normal distribution (mean 0 and variance 1) and *e*_*j*_ is the random measurement error of each item.

Predictive validity was evaluated through a generated criterion variable with normal distribution N(0,1), correlation = 0.7 with the 10-item scale, and dichotomized by an established cut point of *Z* = 1 (*p* = 1 − 0.8413), a simulation situation in which only about 15% of subjects with best scores in the criterion have been selected.

Lack of MI was manipulated replacing progressively discriminant items (Group 1) for items with factor loadings equal to cero for the second sample (Group 2). In other words, Differential Item Functioning (DIF) was introduced progressively on the 10-item scale, so that in these items all variance in the second sample would be attributed to error and, thus, all responses would be entirely random.

A Receiver Operating Characteristic (ROC) curve analysis was used to evaluate the effect of the number of items with DIF on the correct classification in criterion variable. This analysis is a fundamental tool to evaluate predictive validity of psychological tests, since allows to detect cases correctly classified in the criterion and identify the cut point that maximizes sensitivity or true positives and specificity or true negatives (Swets and Pickett, 1982).

## Results

First row of Table 1 shows that, when simulated scale have no DIF, sensibility and specificity both Group 1 and Group 2 are between 0.75 and 0.77. Rest of the rows of Table 1 show the progressive negative effect over sensibility and specificity as the number of DIF items in the scale increases. For example, with 1 DIF item total scale sensitivity is 0.744, with 5 DIF items is 0.710, and with 10 DIF items is 0.605.

**Table 1:** Total, Group 1 and Group 2 sensibility and specificity regarding the number of DIF items manipulated in the simulated 10-item scale.

**Reliability**	**Number of DIF items**	**Total sensitivity**	**Group 1 sensitivity**	**Group 2 sensitivity**	**Total specificity**	**Group 1 specificity**	**Group 2 specificity**
0.769	0	0.749	0.748	0.751	0.763	0.773	0.750
0.745	1	0.744	0.756	0.733	0.760	0.773	0.745
0.720	2	0.740	0.764	0.716	0.751	0.775	0.724
0.696	3	0.736	0.772	0.699	0.743	0.783	0.702
0.670	4	0.726	0.783	0.669	0.739	0.794	0.681
0.645	5	0.710	0.788	0.632	0.726	0.804	0.645
0.622	6	0.698	0.801	0.595	0.711	0.811	0.607
0.604	7	0.678	0.807	0.549	0.689	0.820	0.554
0.591	8	0.655	0.812	0.497	0.668	0.821	0.510
0.585	9	0.632	0.819	0.444	0.648	0.827	0.464
0.585	10	0.605	0.825	0.384	0.624	0.833	0.410

This decrease in sensitivity (and specificity) may seem an acceptable loss of discriminative capacity, although overall results are masking its true effects. It can be observed that both Group 2 sensitivity and specificity values have a more pronounced decrease than that observed in the total results. Conversely, in Group 1 sensitivity and specificity increases as the number of items with DIF increases, which is undetectable when observing total results. Both tendencies are undesired effects of lack of MI.

## Discussion

In this paper we have exposed that the presence of DIF in the items of a scale implies an important violation of the MI of the instrument, and this lack of MI has significant negative effects on predictive validity.

The results show that when reliability of the scale decreases in one of the subsamples (due to the presence of non-discriminating items), the probability that the subjects of this sample exceed the cut point decreases. When this situation occurs, the cut point for the total sample will also decrease and, therefore, subjects of the subsample without DIF will see their options of exceeding the corrected cut point increased.

The loss of discrimination in one or more items from which the lack of MI has been generated is related to non-uniform DIF defined in the Item Response Theory (IRT) framework. Non-uniform DIF usually goes unnoticed as it does not affect the mean of the groups. However, as we have shown in this paper, non-uniform DIF (and consequently, the lack of MI), can have serious consequences when the test is used for predictive or diagnostic purposes. The results imply that one of the two groups (Group 2) would be randomly diagnosed, without any consideration about the real presence of the measured condition, while the other group (Group 1), would be over-diagnosed. Within a selection process, such as an exam, tests scores clearly loses reliability in Group 2 (situation that illegitimately denying the participants any chance of passing the test according to their skills), while increasing those chances on Group 1.

Consequently, researchers should be conscious of the serious implications of using scales and tests that might have non-invariant items when approaching diagnostic and selective processes. It is surprising to find through a simple search that, in the 123,000 studies from 2014 to 2017 that are shown in Google Scholar with the term “gender differences,” only 3.73% does the term “metric invariance.” Despite warnings from psychometricians, research works that regards DIF analysis as an important step in the process of developing a scale are scarce, so it becomes this paper's goal to increase awareness of the necessity and usefulness of such analysis.

## Author contributions

DB-C proposed the project, JA developed the theoretical aspects and DB-C performed computations and analysis under JA's supervision. DO contributed to expand theoretical explanation, as well as interpretation of data. All authors discussed the results and contributed to the final manuscript.

### Conflict of interest statement

The authors declare that the research was conducted in the absence of any commercial or financial relationships that could be construed as a potential conflict of interest.

## Footnotes

^1^It should be noted that, when data does not fit to the parallel model (i.e., equal true scores and equal standard errors), to estimate reliability it is necessary to know error variances in addition to factorial loadings (see Steenkamp and Baumgartner, 1998).
